# Impact of Plant Pre‐Processing on the Chemical Profile of *Psidium guajava* L. (Myrtaceae) Essential Oil and Evaluation of Its Bioactive Potential

**DOI:** 10.1002/cbdv.71843

**Published:** 2026-10-15

**Authors:** Tarsila da C. Silva, Ari Sérgio de O. Lemos, Caio Juan S. Fortes, Débora N. Eiriz, Elaine S. Coimbra, Lucas de A. Carvalho, Luciana M. Chedier, Lucas M. Duarte, Núbia B. Andrade, Rodrigo L. Fabri, Selma R. de Paiva, Alessandra L. Valverde

**Affiliations:** ^1^ Department of Organic Chemistry Federal Fluminense University (UFF) Niterói Rio de Janeiro Brazil; ^2^ Laboratory of Bioactive Natural Products, Department of Biochemistry, Institute of Biological Sciences Federal University of Juiz de Fora (UFJF) Juiz de Fora Minas Gerais Brazil; ^3^ Analytical Separations and Chemometrics Research Group Federal Fluminense University (UFF) Niterói Rio de Janeiro Brazil; ^4^ Department of Parasitology, Microbiology, and Immunology, Institute of Biological Sciences Federal University of Juiz de Fora (UFJF) Juiz de Fora Minas Gerais Brazil; ^5^ Department of Botany, Institute of Biological Sciences Federal University of Juiz de Fora (UFJF) Juiz de Fora Minas Gerais Brazil; ^6^ Department of General Biology, Institute of Biology Federal Fluminense University (UFF) Niterói Rio de Janeiro Brazil

**Keywords:** antifungal activity, anti‐inflammatory activity, *Candida auris*, *Psidium guajava*, β‐caryophyllene

## Abstract

The chemical composition and bioactivity of essential oils depend not only on agronomic and environmental factors but also on the pre‐processing of the plant material, a step that is rarely evaluated. Leaf essential oils of *Psidium guajava* L. were obtained by hydrodistillation from fresh or previously frozen leaves fragmented manually or mechanically, and assessed for GC‐MS composition, cytotoxicity and biological activities. Fresh leaves ground in a mixer (EOFH‐M) gave a sesquiterpene‐rich profile dominated by β‐caryophyllene and α‐humulene, along with the best cell‐viability profile in L929 fibroblasts and BALB/c macrophages. EOFH‐M reduced nitric oxide and intracellular reactive oxygen species and showed a fungicidal effect against *Candida albicans* and the emerging and resistant microorganism *Candida auris*, whereas isolated β‐caryophyllene was fungistatic. Leaf pre‐processing therefore substantially influences the composition, safety and antifungal potential of *P. guajava* essential oil.

## Graphical abstract

Leaf pre‐processing dictates the chemistry and bioactivity of *Psidium guajava* essential oil. Fresh leaves ground in a mixer showed a β‐caryophyllene‐rich sesquiterpene profile and the best safety, together with anti‐inflammatory, antioxidant and fungicidal activity against *Candida* species, including the emerging multidrug‐resistant *Candida auris*, highlighting biomass standardization as a key step. The graphical abstract was created by the authors in Microsoft PowerPoint; no artificial‐intelligence image‐generation tool was used in its creation. The leaf, hand‐fragmentation, and hydrodistillation (Clevenger‐type apparatus) icons are original artwork by the authors. The photograph of *P. guajava* leaves and flower is original work by the authors. The remaining icon elements (Public Domain, courtesy of NIAID) were obtained from the NIAID NIH BioArt Source (bioart.niaid.nih.gov): NIAID Visual & Medical Arts, “128 Well Plate Top View” (BIOART‐000595; BIOART‐000596; Ryan Kissinger), “Conical Sample Tube” (BIOART‐000708; Ryan Kissinger and Austin Athman), “Mass Spectrometer Machine” (BIOART‐000579; BIOART‐000286; Ryan Kissinger), and “Macrophage” (BIOART‐000312; Ryan Kissinger). The scissors icon (“Ring Scissors”, Shuhan He) was obtained from SciDraw (scidraw.io) under a CC‐BY 4.0 license (https://doi.org/10.5281/zenodo.3926081).

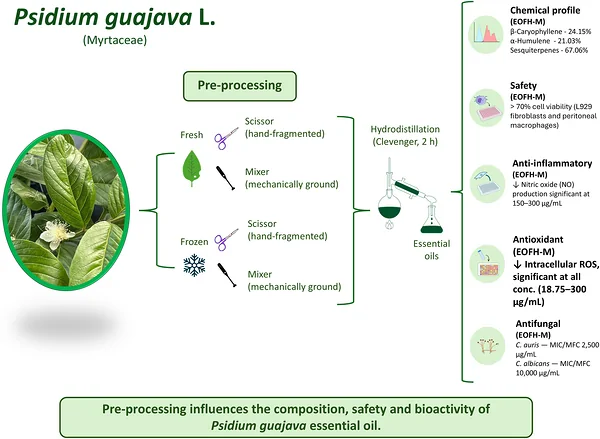

## 1 Introduction

Essential oils are complex mixtures of volatile hydrocarbons, alcohols, terpenoids, and phenylpropanoids produced by aromatic plants as part of their defense and signaling systems and widely explored for their therapeutic potential [1]. Their use is favored by low cost, efficacy, safety and ready availability [2]. Herbal medicines remain central to primary health care for a large part of the population in developing countries [3]. However, the chemical composition and yield of essential oils vary considerably according to species, plant organ, developmental stage, extraction method, and environmental conditions, directly influencing their biological activities [4, 5].

In addition to these factors, pre‐processing of the plant material represents an often‐overlooked source of variation. Procedures such as freezing, drying, and grinding can modify the availability of volatile constituents, influencing both the composition and biological properties of the resulting essential oil [6, 7]. Standardizing these variables is thus a prerequisite for reproducible processes and for a consistent interpretation of phytochemical and biological results [8].

The need for well‐characterized essential oils becomes particularly pressing in antifungal research, an area in which these natural matrices have attracted growing interest [3]. Among the pathogens for which new agents are urgently required are yeasts of the genus *Candida*, opportunistic microorganisms responsible for clinical manifestations ranging from superficial infections to potentially severe systemic disease, especially in immunocompromised individuals [9, 10]. *Candida albicans* is the most frequently species associated with mucocutaneous and invasive candidiasis, whereas *Candida auris* has emerged as a global public‐health threat owing to its multidrug resistance, environmental persistence and capacity for hospital dissemination [11, 12]. In 2022, the World Health Organization included both species in its first priority list of fungal pathogens [13]. As resistance to the limited arsenal of conventional antifungals continues to rise [14, 15], natural products, and essential oils in particular, stand out as promising sources of new bioactive substances, combining antifungal, antioxidant and anti‐inflammatory constituents [16, 17].

Among the plant species explored for this purpose, *Psidium guajava* L. (Myrtaceae), the guava tree, is especially promising. Native to South America and widely distributed across tropical regions and Brazilian biomes [18], it has been listed, since 2009, in the Brazilian National List of Medicinal Plants of Interest to the Unified Health System (RENISUS) [19]. Its leaves are traditionally used as infusions or decoctions to manage diabetes, gastrointestinal and inflammatory disorders, vaginal problems and pain, and their activity against pathogenic microorganisms has been repeatedly documented [20, 21]. From an ethnobotanical standpoint, *P. guajava* is widely used medicinal plants in tropical and subtropical regions, and virtually all of its organs are employed in traditional medicine across Latin America, Africa and Asia. In addition to the applications mentioned above, leaf decoctions and infusions are popularly used to treat diarrhea, dysentery, wounds, ulcers, coughs, toothache and febrile conditions, whereas the fruit is valued for its nutritional and vitamin content. This rich ethnomedicinal record has long guided the pharmacological investigation of the species and remains a valuable rationale for characterizing its essential oil [18, 20]. The leaf essential oil is rich in sesquiterpenes, with β‐caryophyllene frequently reported as a major constituent [22], a compound credited with a broad range of biological properties, including anti‐inflammatory, analgesic and antimicrobial effects [23, 24].

Although the chemistry and bioactivity of *P. guajava* leaf essential oil are well documented, most studies characterize the oil obtained from a single biomass‐preparation protocol, and the influence of leaf pre‐processing has rarely been addressed in an integrated way. Accordingly, the present work investigated whether different leaf pre‐processing conditions, fresh or previously frozen material, combined with manual or mechanical fragmentation, affect the yield, chemical profile and cytotoxicity of the essential oil, in order to select the oil with the best characteristics. The selected oil was then evaluated for its anti‐inflammatory, antioxidant effects, and antifungal potential against *Candida* species.

## 2 Results and Discussion

### 2.1 Essential Oil Chemical Composition

All essential oils displayed a similar qualitative profile, characterized by a predominance of sesquiterpenes and smaller proportions of monoterpenes and oxygenated terpenoids (Table 1 and Figure 1). β‐Caryophyllene and α‐humulene were the major constituents in all samples, in agreement with previous reports for *P. guajava* leaf essential oil [2]. However, the chromatographic profiles revealed variations in the relative distribution of constituents according to the pre‐processing conditions applied to the leaves, including their presentation prior to extraction (fresh or frozen) and the fragmentation method. Notably, when identical GC‐MS acquisition and peak‐integration parameters were applied, oils obtained from mixer‐ground leaves yielded a greater number of identified constituents than those obtained from leaves fragmented with scissors, suggesting that more extensive tissue disruption may favor the detection of minor volatile compounds. In addition, because the same mass of plant material was subjected to hydrodistillation and analyzed under identical GC‐MS conditions, the chromatograms obtained from fresh leaves generally exhibited higher absolute peak areas for major constituents such as β‐caryophyllene, α‐humulene, limonene, and 1,8‐cineole than those obtained from frozen leaves. Although this observation suggests a greater recovery of volatile constituents from fresh biomass, these differences should be interpreted with caution, as GC‐MS peak areas do not provide accurate quantitative information. Confirmation would require quantitative GC‐FID analyses using validated calibration curves, which were beyond the scope of the present study.

**TABLE 1:** Chemical composition (relative peak area, %) of the leaf essential oils of *Psidium guajava* L. obtained under four pre‐processing conditions, determined by GC‐MS.

Compound	RI (exp.)	RI (lit.)	SI (%)	Area (%) EOFH‐M	Area (%) EOFH‐S	Area (%) EOFZ‐M	Area (%) EOFZ‐S
α‐Pinene	934	932	86	0.27	0.15	n.d.	n.d.
Benzaldehyde	962	952	92	0.29	1.06	0.15	1.19
Myrcene	988	988	77	0.08	n.d.	n.d.	n.d.
Limonene	1030	1024	92	6.89	9.66	4.01	8.78
1,8‐Cineole	1033	1026	94	5.97	11.77	4.26	5.06
α‐Terpineol	1196	1186	76	0.13	n.d.	0.08	n.d.
α‐Copaene	1377	1374	75	0.18	n.d.	0.08	n.d.
β‐Caryophyllene	1421	1417	96	24.15	21.27	19.66	14.42
α‐Humulene	1457	1452	93	21.03	22.62	24.15	34.12
4,5‐di‐epi‐Aristolochene	1472	1471	—	0.11	n.d.	0.05	n.d.
γ‐Muurolene	1475	1478	—	0.81	n.d.	0.44	n.d.
β‐Selinene	1491	1489	—	10.63	7.38	8.39	4.41
α‐Selinene	1498	1498	92	9.67	5.88	7.26	4.02
δ‐Cadinene	1520	1522	70	0.14	n.d.	0.03	n.d.
trans‐Dauca‐4(11),7‐diene	1556	1556	—	0.34	n.d.	0.35	n.d.
(E)‐Nerolidol	1560	1561	88	1.37	1.17	1.62	1.75
Caryophyllene oxide	1584	1582	—	2.88	2.40	4.27	2.34
Humulene epoxide II	1612	1608	—	1.29	0.55	2.69	4.13
1,10‐di‐epi‐Cubenol	1619	1618	—	0.61	n.d.	1.02	n.d.
allo‐Aromadendrene epoxide	1635	1639	—	1.82	1.21	4.02	4.90
Selina‐3,11‐dien‐6α‐ol	1639	1642	—	2.44	1.92	4.50	1.59
α‐Muurolol (Torreyol)	1648	1644	—	0.54	0.27	0.85	n.d.
Selin‐11‐en‐4α‐ol	1660	1658	—	7.17	11.95	10.19	9.33
**∑ Monoterpenes**				7.24	9.81	4.01	8.78
**∑ Sesquiterpenes**				67.06	57.15	60.41	56.97
**∑ Oxygenated terpenoids**				24.22	31.24	33.50	29.10
**Total Identified (%)**				**98.47**	**99.26**	**98.07**	**96.04**

EOFH‐M/EOFH‐S: fresh leaves ground in a mixer (M)/fragmented with scissors (S); EOFZ‐M/EOFZ‐S: previously frozen leaves ground in a mixer (M)/fragmented with scissors (S). RI (exp.): experimental retention index relative to a homologous series of *n*‐alkanes (C7–C40), representative value across samples; RI (lit.): literature retention index; n.d.: not detected. Constituents are listed in elution order; minor unidentified peaks (each ≤ 1.5%) are not listed.

**FIGURE 1:**
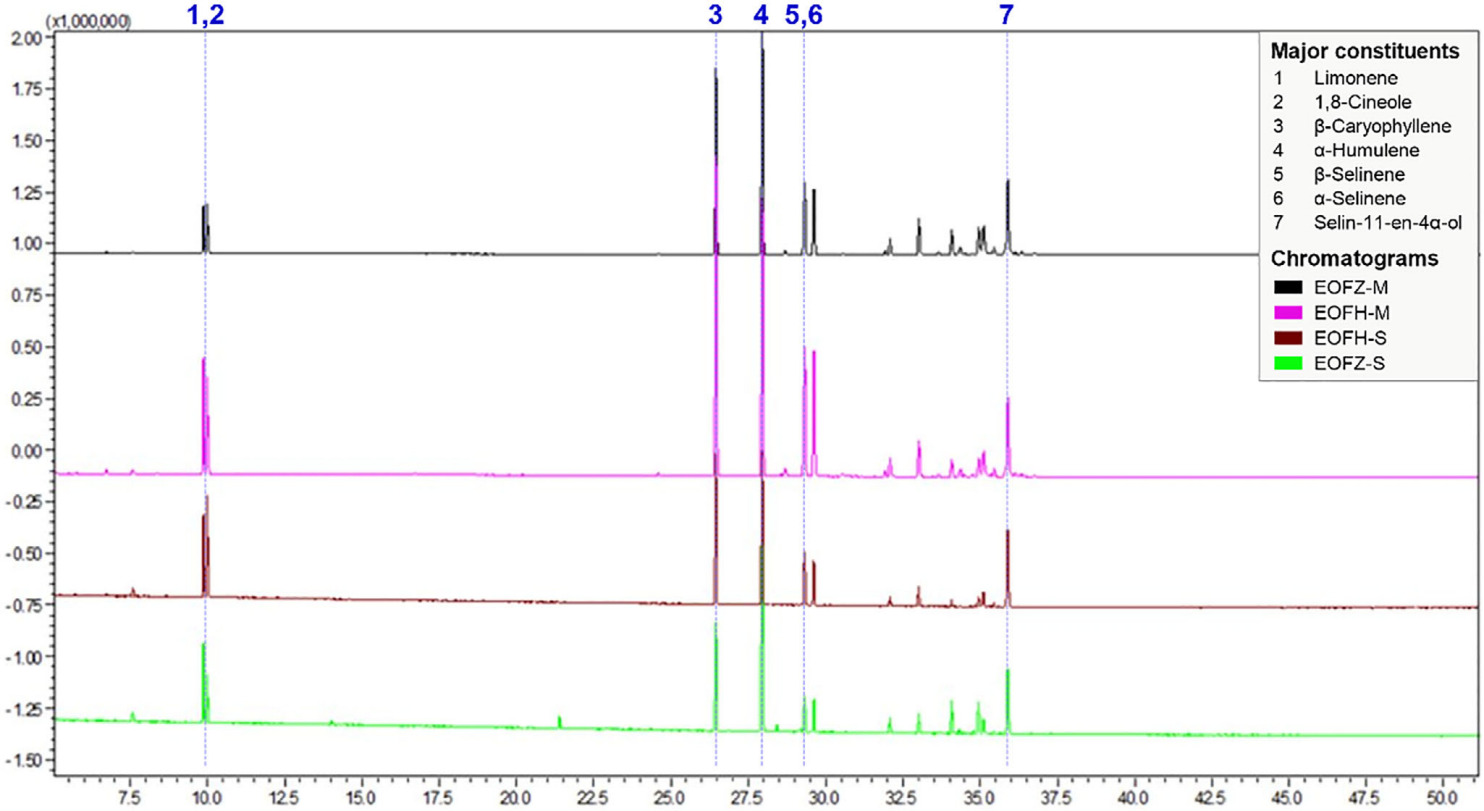
GC‐MS chromatograms of the leaf essential oils of *Psidium guajava* L. obtained under the four pre‐processing conditions: (1) EOFZ‐M (frozen leaves, mixer); (2) EOFH‐M (fresh leaves, mixer); (3) EOFH‐S (fresh leaves, scissors); (4) EOFZ‐S (frozen leaves, scissors).

These differences may be associated with changes in tissue integrity before hydrodistillation. Mechanical grinding is likely to promote more extensive rupture of secretory structures [25], whereas fragmentation with scissors results in a gentler disruption of the plant matrix. Likewise, freezing may alter cellular organization through ice‐crystal formation, potentially affecting the release and recovery of volatile constituents during extraction [26, 27]. Similar effects of post‐harvest handling and sample preparation, including storage conditions and grinding intensity, have been reported for other aromatic species, highlighting the influence of biomass processing on essential‐oil composition [28, 29, 30]. The dependence of essential‐oil yield and composition on the extraction procedure and on post‐harvest handling has likewise been documented for other aromatic species, notably *Rosmarinus officinalis* L., reinforcing the importance of standardizing every step that precedes hydrodistillation [31, 32]. Overall, the results suggest that pre‐processing conditions can influence the chemical profile of *P*. *guajava* essential oil and should therefore be carefully standardized in phytochemical and biological studies.

### 2.2 Cytotoxicity and Selection of the Pre‐Processing Condition

Biological safety was assessed in L929 fibroblasts and BALB/c peritoneal macrophages by the MTT assay after 48 h of exposure (18.75–300 µg/mL). In L929 fibroblasts, essential oils obtained from fresh leaves preserved cell viability more effectively than those extracted from frozen leaves. EOFH‐M and EOFH‐S maintained viability close to the control throughout the entire concentration range tested, whereas oils from frozen leaves, especially EOFZ‐M, caused the most pronounced decrease in viability at 300 µg/mL (Figure 2).

**FIGURE 2:**
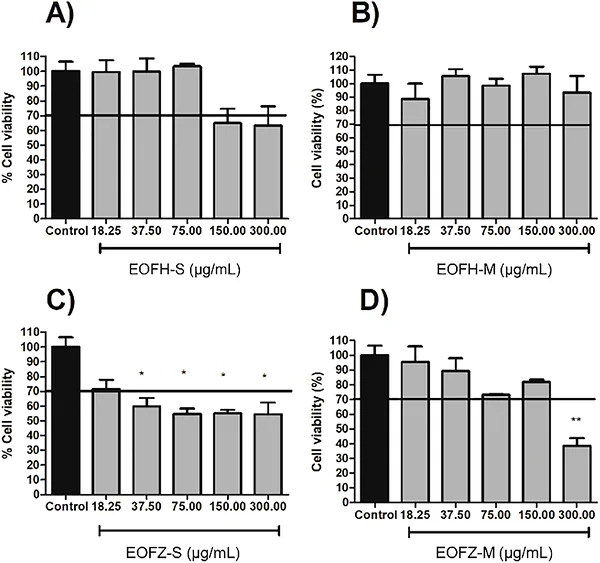
Cell viability of L929 murine fibroblasts after 48 h of exposure to the leaf essential oils of *Psidium guajava* L. obtained under different pre‐processing conditions, determined by the MTT assay. Panels A–D correspond to EOFH‐S, EOFH‐M, EOFZ‐S and EOFZ‐M, respectively (concentrations in µg/mL); the horizontal line marks 70% viability. Data are expressed as mean ± SD; differences versus the negative control were assessed by one‐way ANOVA followed by Bonferroni's post hoc test (*n* = 3, performed in triplicate; GraphPad Prism 5.0); *p < 0.05 and **p < 0.01 versus the negative control. Essential oils obtained from fresh leaves fragmented with scissors (EOFH‐S) or ground in a mixer (EOFH‐M). Essential oils obtained from previously frozen leaves fragmented with scissors (EOFZ‐S) or ground in a mixer (EOFZ‐M).

A comparable pattern was observed in BALB/c peritoneal macrophages: the oils from fresh leaves, and particularly EOFH‐M, maintained viability above 70% throughout the tested range, while the oils from frozen leaves showed again the strongest cytotoxic effect at the highest concentration, EOFZ‐M reducing viability to about half of the control at 300 µg/mL (Figure 3).

**FIGURE 3:**
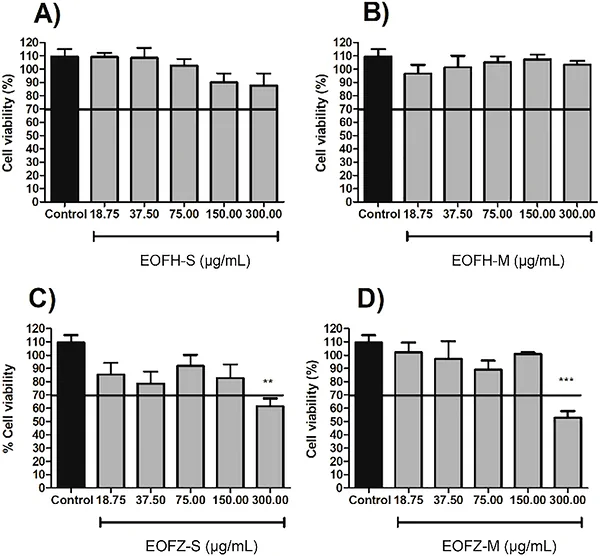
Cell viability of BALB/c peritoneal macrophages after 48 h of exposure to the leaf essential oils of *Psidium guajava* L. obtained under different pre‐processing conditions, determined by the MTT assay. Panels A–D correspond to EOFH‐S, EOFH‐M, EOFZ‐S, and EOFZ‐M, respectively (concentrations in µg/mL); the horizontal line marks 70% viability. Data are expressed as mean ± SD; differences versus the negative control were assessed by one‐way ANOVA followed by Bonferroni's post hoc test (*n* = 3, performed in triplicate; GraphPad Prism 5.0); *p < 0.05, **p < 0.01, and ***p < 0.001 versus the negative control. Essential oils obtained from fresh leaves fragmented with scissors (EOFH‐S) or ground in a mixer (EOFH‐M). Essential oils obtained from previously frozen leaves fragmented with scissors (EOFZ‐S) or ground in a mixer (EOFZ‐M).

Thus, freezing not only altered the chemical composition but also increased cytotoxicity, reinforcing the need for rigorous standardization of the steps preceding hydrodistillation. The greater cytotoxicity exhibited by the oils obtained from frozen leaves was accompanied by a higher relative content of oxygenated terpenoids (Table 1), suggesting that the compositional shifts induced by freezing may underlie the reduced cell viability, although the specific contribution of individual constituents remains to be determined. Considering qualitative chemical profile and safety together, EOFH‐M was selected for the subsequent biological and antifungal assays.

### 2.3 Anti‐Inflammatory Activity

The anti‐inflammatory potential of EOFH‐M was evaluated through nitric oxide (NO) production in LPS‐ and IFN‐γ‐stimulated peritoneal macrophages (Figure 4). No significant change was observed at 18.75–75 µg/mL, whereas a significant reduction occurred at 150 µg/mL and the strongest inhibition at 300 µg/mL, indicating a concentration‐dependent down‐modulation of NO production in activated macrophages. Because excessive NO is a key effector of the inflammatory response, this effect points to a potential modulatory role of the oil, in agreement with the anti‐inflammatory properties attributed to β‐caryophyllene‐rich oils [23].

**FIGURE 4:**
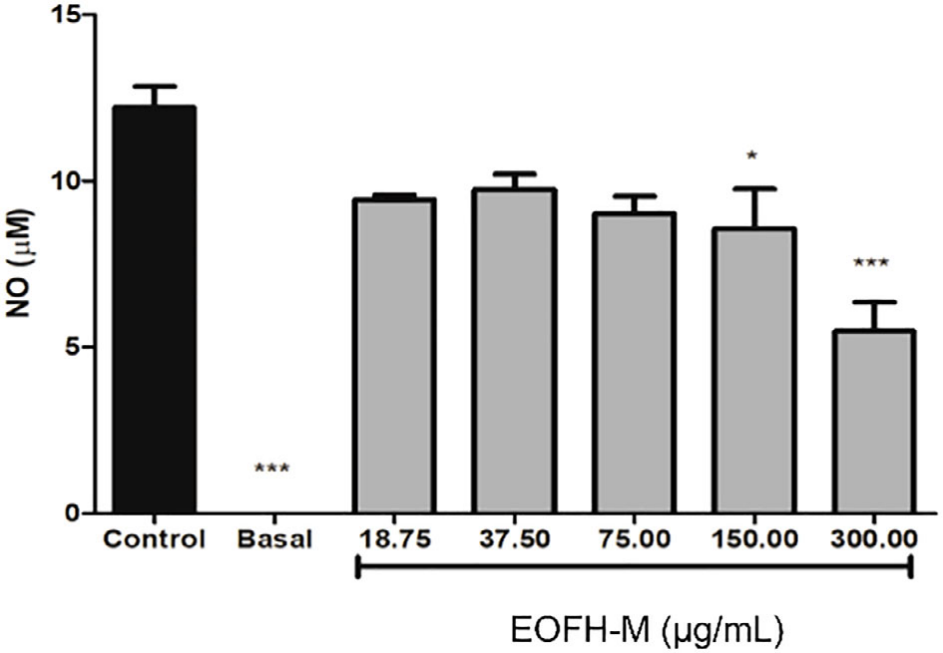
Nitric oxide (NO) production in LPS‐ and IFN‐γ‐stimulated peritoneal macrophages treated with different concentrations of the essential oil of *Psidium guajava* L. obtained from fresh leaves ground in a mixer (EOFH‐M). Control: cells stimulated with LPS (1 µg/mL) and IFN‐γ (1 ng/mL) without oil treatment; Basal: unstimulated, untreated cells. Data are expressed as µM NO (mean ± SD); differences versus the control were assessed by one‐way ANOVA followed by Bonferroni's post hoc test using GraphPad Prism 5.0 (*n* = 3, performed in triplicate; *p < 0.05 and ***p < 0.001 versus the control).

### 2.4 Antioxidant Activity

Intracellular reactive oxygen species (ROS) were quantified in stimulated macrophages using the fluorescent probe H2DCFDA (Figure 5). EOFH‐M significantly reduced ROS levels at all tested concentrations (18.75–300 µg/mL) relative to the stimulated control, with no significant differences among concentrations, suggesting a non‐linear, possibly saturable response consistent with synergistic action among constituents [33]. Combined antimicrobial, anti‐inflammatory and antioxidant effects have similarly been reported for other plant‐derived extracts, reinforcing the relevance of a multi‐target bioactivity profile [34]. Antioxidant and antimicrobial activities have likewise been reported for other terpene‐rich essential oils, such as those of *Lavandula angustifolia* Mill. (sin. *Lavandula officinali* Chaix) and several Mediterranean aromatic species [35, 36]. In the chemical assays (Table 2), the oil showed low activity in the DPPH^•^ assay (IC_50_> 250 µg/mL versus 2.35 µg/mL for rutin) but a marked reducing capacity in the phosphomolybdenum assay (194.08% relative to rutin) and moderate protection in the β‐carotene/linoleic acid system (18.50% inhibition at 38.46 µg/mL). The weak DPPH^•^ response is typical of terpene‐rich oils, which are less efficient as hydrogen donors in the absence of phenolic groups and whose lipophilicity hampers interaction with the radical in polar media [37, 38], whereas the strong phosphomolybdenum response indicates that electron‐transfer mechanisms predominate, in line with the redox activity reported for sesquiterpenes such as β‐caryophyllene and α‐humulene [39].

**FIGURE 5:**
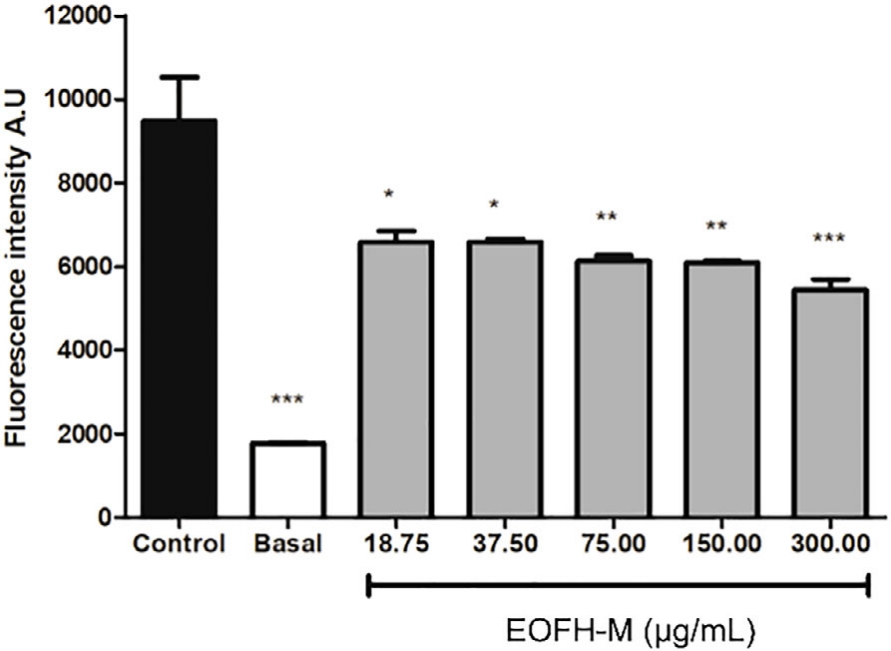
Intracellular reactive oxygen species (ROS) in LPS‐ and IFN‐γ‐stimulated peritoneal macrophages treated with the essential oil of *Psidium guajava* L. obtained from fresh leaves ground in a mixer (EOFH‐M), expressed as fluorescence intensity (arbitrary units). Control (positive control): cells stimulated with LPS (1 µg/mL) and IFN‐γ (1 ng/mL) without oil treatment; Basal: unstimulated, untreated cells. Differences versus the positive control were assessed by one‐way ANOVA followed by Bonferroni's post hoc test using GraphPad Prism 5.0 (*n* = 3, performed in triplicate). Data are expressed as mean ± SD; *p < 0.05, **p < 0.01, and ***p < 0.001 versus the positive control.

**TABLE 2:** Antioxidant activity of the essential oil of *Psidium guajava* L. obtained from fresh leaves ground in a mixer (EOFH‐M) determined by the DPPH^•^, phosphomolybdenum and β‐carotene/linoleic acid assays.

Samples	DPPH • —IC_50_ (µg/mL)	Phosphomolybdenum complex reduction		β‐Carotene/linoleic acid (38.46 µg/mL)		
		% relative of rutin	% relative of quercetin	Inhibition (%)	F1	F2
*EOFH‐M*	>250*	194.08 ± 7.22*	91.01 ± 3.37	18.50 ± 4.11*	0.82± 0.03*	1.04± 0.10*
Rutin	2.35 ± 0.11	—	—	64.586 ± 1.333	0.241 ± 0.046	0.575 ± 0.044

IC_50_: concentration required to scavenge 50% of DPPH^•^ radicals; β‐carotene/linoleic acid inhibition determined at 38.46 µg/mL; –: not applicable. DPPH, 2,2‐diphenyl‐1‐(2,4,6‐trinitrophenyl)hydrazyl; F1, relationship between the tangent values of the sample and negative control between 15 and 45 min; F2, relationship between the sample values and the negative control between 75 and 90 min. *Statistical difference between EOFH‐M and rutin. Statistical analysis was performed using Student's *t*‐test (*p < 0.05). Results are expressed as mean ± SD of three independent experiments performed in triplicate (*n* = 3).

### 2.5 Antifungal Activity

The antifungal activity of EOFH‐M and of β‐caryophyllene, its major constituent, was determined against *C. albicans* ATCC 24433, *C. albicans* ATCC 10231, a clinical vulvovaginal isolate of *C. albicans* and *C. auris* CDC B11903, with nystatin as positive control (Table 3). EOFH‐M was active against all strains, with MIC = MFC = 10,000 µg/mL for the three *C. albicans* strains and MIC = MFC = 2,500 µg/mL for *C. auris*, indicating a fungicidal effect under the tested conditions. β‐Caryophyllene reproduced the same MIC values but did not reach the MFC at the tested concentrations, displaying a predominantly fungistatic profile.

**TABLE 3:** Minimum inhibitory concentration (MIC) and minimum fungicidal concentration (MFC) of the essential oil of *Psidium guajava* L. obtained from fresh leaves ground in a mixer (EOFH‐M) and of β‐caryophyllene against *Candida* spp.

Strain	EOFH‐M MIC (µg/mL)	EOFH‐M MFC (µg/mL)	β‐Caryophyllene MIC (µg/mL)	β‐Caryophyllene MFC (µg/mL)	Nystatin MIC (µg/mL)
*C. albicans* ATCC 24433	10 000	10,000	10 000	>10 000	2.5
*C. albicans* ATCC 10231	10 000	10,000	10 000	>10 000	2.5
*C. albicans* (vulvovaginal clinical isolate)	10 000	10,000	10 000	>10 000	2.5
*C. auris* CDC B11903	2500	2500	2500	>2500	1.25

Nystatin was used as the positive control. Assays were performed in duplicate (*n* = 2). MIC, minimum inhibitory concentration; MFC, minimum fungicidal concentration (values in µg/mL); MIC = MFC indicates a fungicidal effect, whereas MFC > MIC indicates a fungistatic effect.

The lower MIC against *C. auris* is particularly relevant given the emerging and multidrug‐resistant nature of this species, for which data on *P. guajava* oil are still scarce [40]. Essential oils act on multiple cellular targets and cause direct membrane damage, which may be advantageous against resistance mechanisms such as ergosterol‐pathway alterations, biofilm formation and efflux systems described for *C. auris* [41]. The terpenoid mode of action is mainly related to lipophilicity‐driven interaction with the fungal membrane, altering permeability, disrupting ergosterol and impairing osmotic and metabolic homeostasis [42]. The MIC values obtained for *C. albicans* were comparable to those reported for a β‐caryophyllene‐rich *P. guajava* oil [43] and higher than the concentrations required for crude guava extracts [44]. The contrast between the fungicidal activity of the oil and the fungistatic effect of isolated β‐caryophyllene suggests that the antifungal activity may result from synergistic or additive interactions within the phytocomplex rather than from a single compound [45]. β‐Caryophyllene may additionally induce oxidative stress and morphological changes in yeasts [46], while other constituents such as caryophyllene oxide, limonene, nerolidol and 1,8‐cineole may act in a complementary way on different cellular targets [47]. Although the required concentrations are relatively high, a common feature of crude essential oils owing to their physicochemical properties [48], the profile remains pharmacologically relevant, and nanoemulsification has been proposed to improve the solubility, stability and antimicrobial performance of such oils [49].

## 3 Conclusions

By systematically linking four leaf pre‐processing conditions to chemical composition, cytotoxicity and a multi‐target bioactivity profile (anti‐inflammatory, antioxidant and antifungal) within a single experimental framework, this study offers an integrated perspective seldom addressed in the essential oil literature, where such variables are typically examined in isolation. Leaf pre‐processing, often treated as a merely operational step, proved to be a decisive determinant of the chemical and biological characteristics of *P. guajava* essential oil. Among the conditions tested, fresh leaves ground in a mixer (EOFH‐M) combined a greater number of identified constituents than those fragmented with scissors, a sesquiterpene‐dominated profile rich in β‐caryophyllene and the most favorable safety profile, whereas freezing the biomass reduced the recovery of volatile constituents and enhanced cytotoxicity. EOFH‐M exhibited a concentration‐dependent inhibition of NO production, significant reduction of intracellular ROS and a fungicidal effect against all *Candida* strains, including the emerging *C. auris*, against which it showed the greatest activity. The fungistatic behavior of isolated β‐caryophyllene suggests a synergistic action within the phytocomplex. Overall, these findings demonstrate that standardizing biomass preparation is essential for obtaining reproducible, safe and bioactive essential oils, and they highlight *P. guajava* oil as a promising source of antifungal agents against clinically relevant *Candida* species.

## 4 Experimental Section

### 4.1 Plant Material and Pre‐Processing

Leaves of *P. guajava* L. (Myrtaceae) were collected in April 2025 from Comendador Levy Gasparian, Rio de Janeiro, Brazil (22°03′03.4″S, 43°12′40.4″W; ca. 315 m a.s.l.), at intermediate canopy height (ca. 1.9 m), between 07:30 and 08:00 a.m. The botanical material was identified by Dr. André Hoffmann Pereira Pinto (Department of General Biology, Institute of Biology, Fluminense Federal University), and a voucher specimen was deposited in the Herbarium of Niterói, Fluminense Federal University (NIT 17894), and access to genetic heritage was registered in the Brazilian National System for the Management of Genetic Heritage and Associated Traditional Knowledge (SisGen, ABFEA6F). For the comparative study, 600 g of fresh leaves were divided into four 150 g portions submitted to different pre‐processing methods: fresh leaves fragmented with scissors (EOFH‐S), fresh leaves ground in a mixer (EOFH‐M), previously frozen at –20°C leaves fragmented with scissors (EOFZ‐S) or ground in a mixer (EOFZ‐M).

### 4.2 Essential Oil Extraction and Yield

Essential oils were obtained by hydrodistillation for 2 h in a Clevenger‐type apparatus, according to the Brazilian Pharmacopoeia [50]. Preliminary assays used 150 g of leaves and 1000 mL of reverse‐osmosis water. Yield was calculated as the ratio between the mass of essential oil obtained and the mass of plant material [51].

### 4.3 Chemical Characterization (GC‐MS)

The chemical composition of the essential oil was analyzed by gas chromatography–mass spectrometry (GC–MS) using a Shimadzu GC‐2010 gas chromatograph coupled to a QP2010 Ultra mass spectrometer, equipped with an AOC‐20i autosampler. Separations were performed on an SPB‐5MS fused‐silica capillary column (30 m × 0.25 mm i.d., 0.25 µm film thickness; Supelco, Bellefonte, PA, USA). Samples were injected in split mode (20:1) with the injector temperature set at 260°C. Helium was used as the carrier gas at a constant flow rate of 1.0 mL min^−^^1^. The oven temperature program was set from 60°C to 290°C at a rate of 3°C min^−^^1^. The ion source temperature was maintained at 230°C, while the interface temperature was 290°C. Mass spectra were obtained in electron ionization (EI) mode at 70 eV, operating in scan mode over a mass range of *m*/*z* 40–400. The solvent delay time was 3 min. Identification of the components was carried out by comparison of their mass spectra with those from the NIST and Wiley spectral libraries, as well as by comparison of their retention indices (RI) with literature data. Retention indices were calculated relative to a homologous series of *n*‐alkanes (C7–C40) analyzed under the same chromatographic conditions.

### 4.4 Animals

Experiments were performed with male BALB/c mice weighing 20–25 g at 30 days of age obtained from the Federal University of Juiz de Fora Reproduction Biology Center. National Institutes of Health's Ethical Guidelines for the Care and Use of Laboratory Animals were closely followed throughout this study. All procedures were carried out according to institutional policies on animal use in research. The Federal University of Juiz de Fora Committee on the Ethics of Animal Experiments approved the study (Protocol Number: 019/2026‐CEUA).

### 4.5 Cytotoxicity on Mammalian Cells

Macrophages (2 × 10^5^ cells/well), obtained from the peritoneal cavity of BALB/c mice after 72 h of inoculation with 2% thioglycolate, were seeded in 96‐well microplates and cultured for 24 h in RPMI‐1640 medium supplemented with 2 mM L‐glutamine, 5% fetal bovine serum (FBS) and 1% antibiotic solution (penicillin 100 µg/mL and streptomycin 100 µg/mL). The L929 fibroblast cell line (NCTC clone 929; ATCC CCL‐1; RRID:CVCL_0462; American Type Culture Collection, Manassas, VA, USA) was cultured in Dulbecco's Modified Eagle Medium (DMEM) supplemented with 10% FBS and the same antibiotic solution, at a seeding density of 5 × 10^3^ cells per well. Both cell cultures were maintained at 37°C in a humidified atmosphere containing 5% CO_2_ until the experiments were performed. The cells were then exposed to EOFH‐S, EOFH‐M, EOFZ‐S, and EOFZ‐M at doses varying between 18.75 and 300.00 µg/mL. Cells were treated with 0.06% DMSO (vehicle) only as the control. The cells were incubated at 37°C in an environment containing 5% CO_2_ for 48 h. The MTT assay was used to quantify cytotoxicity based on cell viability [52]. At 570 nm, absorbance was measured. The experiment was done in triplicate. Cytotoxicity assessment, as well as the anti‐inflammatory and antioxidant assays, were performed in collaboration with the Instituto de Ciências Biológicas (ICB), Universidade Federal de Juiz de Fora (UFJF).

After initial screening, the best method for extracting essential oil from *P. guajava* was used to conduct anti‐inflammatory, antioxidant, and antifungal tests. The selection criteria were based on chemical diversity and cell toxicity.

### 4.6 Evaluation of Anti‐Inflammatory Activity—NO Dosage

The Griess method (an indirect NO measurement by nitrite dosage) [53, 54] was used to measure NO production. After 48 h of incubation with EOFH‐M (18.75–300.00 µg/mL) and stimulation with LPS at 1 µg/mL and IFN‐γ at 1 ng/mL, nitrite levels were assessed in the macrophage culture supernatants. 48 h were spent incubating the cells at 37°C with 5% CO_2_. Spectrophotometric measurements were taken at 540 nm, and the experiment was conducted in triplicate.

### 4.7 Evaluation of Antioxidant Activity

#### 4.7.1 Reactive Oxygen Species (ROS) Production in Macrophages Stimulated With LPS and IFN‐γ

Following a 48‐hour period, macrophages activated with LPS and IFN‐γ (2 × 10^5^ cells per well) and treated with EOFH‐M at 18.75–300.00 µg/mL had their ROS levels measured. The cells were then labeled with 1 mM of 2,7‐Dichlorofluorescin diacetate (H2DCFDA) (Invitrogen, Waltham, MA, USA) [55]. Using a fluorimeter (FL x 800) at λex/λem = 485/528 nm, fluorescence values were obtained after 30 min, and the experiment was conducted in triplicate. A positive control group of cells was treated with only LPS and IFN‐γ.

### 4.8 Chemical Antioxidant Assays

#### 4.8.1 Evaluation of Antioxidant Activity 2,2‐Diphenyl‐1‐picrylhydrazyl (DPPH^•^) Radical‐Scavenging Method

The free radical‐scavenging activity of DPPH^•^ was determined as described by Brand‐Williams [56]. Successive dilutions of EOFH‐M were performed in 96‐well plates and the 20 µg/mL of DPPH solution in methanol was added, thus obtaining EOFH‐M concentrations ranging from 250 to 0.49 µg/mL. The plate was incubated in the dark and after 30 min the absorbance was measured at 517 nm. The test was performed in triplicate and the percentage (%) of EOFH‐M oxidation inhibition was calculated as follows: % Inhibition = [(AU DPPH − AU sample)/AU DPPH] × 100. Where: AU DPPH: Measured absorbance for DPPH radical in methanol; AU sample: measured absorbance of the samples. The positive control used was rutin, whose concentration ranged from 250 to 0.0005 µg/mL. The results were expressed in the form of 50% inhibitory concentration (IC_50_). The experiment was performed in triplicate.

#### 4.8.2 Total Antioxidant Capacity by Phosphomolybdenum Assay

The total antioxidant capacity (TAC) was determined by phosphomolybdenum assay described by Prieto [57]. Test tubes containing 300 µL of EOFH‐M/positive controls (quercetin and rutin) in ethanolic solution (500 µg/mL) and 2 mL reagent solution (1.12 mL sodium phosphate solution, 0.48 mL ammonium molybdate and 0.40 mL H_2_SO_4_) were heated at 95°C for 90 min. The absorbance of the treated samples was recorded by UV spectroscopy at 695 nm. Results were expressed as a percentage of relative antioxidant activity (RAA%) in relation to the positive controls. The equation for calculating RAA% is: RAA% = [(AU Sample − AU blank of sample)/AU positive control − AU blank of positive control] × 100. Where: AU sample: absorbance measured for the samples; AU positive control: absorbance measured for the positive controls; AU blank of sample: absorbance measured for the sample blank; AU blank of positive control: absorbance measured for the positive control blank. The experiment was performed in triplicate.

#### 4.8.3 β‐Carotene/Linoleic Acid System

Lipid peroxidation inhibition activity was evaluated by the β‐carotene/linoleic acid method described by Melo & Mancini Filho [58] with some modifications. An emulsion containing β‐carotene and linoleic acid was prepared and adjusted for a reading between 0.6 and 0.7 of absorbance at 470 nm. 250 µL of emulsion and 10 µL of EOFH‐M and rutin (positive control) solutions (final concentration of 38.46 µg/mL) were added. A negative control was prepared with 250 µL of this emulsion and 10 µL of methanol (100% oxidation). The plate was incubated at 45°C to accelerate oxidation reactions and initiate β‐carotene discoloration. The absorbance of the samples was measured at 470 nm. Readings were taken at t = 0 and in intervals of 15 min until completion of the reaction after 120 min.

The percentage of lipoperoxidation inhibition (% I) was determined by measuring the decrease in sample absorbance (AU Sample) relative to the negative control (AU Control), according to the formula: % I = (AU Control − AU Sample) × 100/ AU Control. The oxidation curve between the negative control and EOFH‐M/Rutin was evaluated to find the values of F1 (blocking capacity of peroxide formation) between 15 and 45 min after the beginning of the reaction and F2 (capacity of inhibiting other reactions during the oxidative process, which produce radical species) between 75 and 90 min after the beginning of the reaction by the following formulas: F1 = tg solution AU Sample/tg AU Control; F2 = tg solution AU Sample/tg AU Control. Where: AU Control: absorbance measured for negative control; AU Sample: absorbance measured for the sample; % I: percentage of lipoperoxidation inhibition; Tg: tangent. The entire experiment was performed in triplicate.

### 4.9 Antifungal Activity

#### 4.9.1 Minimum Inhibitory Concentration (MIC) Determination

Antifungal susceptibility testing was performed by broth microdilution according to the protocols of the Clinical and Laboratory Standards Institute (CLSI) [59, 60] for determination of the minimum inhibitory concentration (MIC) [59, 60]. The strains *C. albicans* ATCC 24433, *C. albicans* ATCC 10231, a clinical vulvovaginal isolate of *C. albicans* and *C. auris* CDC B11903 were grown on Sabouraud dextrose agar (SDA) and incubated at 35°C for 24 h. The samples, including the essential oils and β‐caryophyllene (Sigma‐Aldrich), were dissolved in Roswell Park Memorial Institute‐1640 medium (RPMI‐1640) containing 1% DMSO (v/v) at a 9:1 ratio, yielding stock solutions at 25 000 µg/mL. Aliquots of 100 µL of RPMI‐1640 were dispensed into 96‐well microplates; 80 µL of the sample stock solution were then added, followed by serial dilutions to obtain concentrations ranging from 10 000 to 78.12 µg/mL. The inoculum was prepared from a fungal suspension containing 10^4^ colony‐forming units per milliliter (CFU/mL), standardized to 1 on the McFarland turbidity scale; 20 µL of this inoculum were added to each well, giving a final concentration of 2 × 10^3^ CFU/mL. The growth control consisted of RPMI‐1640 with 1% DMSO (vehicle) and the microorganism, and an experimental blank containing only RPMI‐1640 and the corresponding sample concentrations (without inoculum) was also prepared. Nystatin was used as the positive control. After incubation at 35°C for 24 h, fungal growth was assessed visually from the turbidity of the medium; wells with a clear medium were considered indicative of the absence of microbial growth. The MIC was defined as the lowest sample concentration that visually prevented fungal growth. All experiments were performed in duplicate.

#### 4.9.2 Minimum Fungicidal Concentration (MFC) Determination

The fungicidal/fungistatic concentrations of the samples, including the essential oils and β‐caryophyllene (Sigma‐Aldrich), were determined using a modification of the method described by Spencer and Ragout de Spencer [61]. After the MIC assay, a 20 µL aliquot from each well showing no fungal growth was withdrawn from the microplates and streaked onto Sabouraud dextrose agar (SDA) plates, which were then incubated at 35°C for 24 h. The minimum fungicidal concentration (MFC) was defined as the lowest concentration able to prevent the growth of fungal colonies on the agar plates. The assay was performed in duplicate.

### 4.10 Statistical Analysis

Quantitative data are expressed as mean ± standard deviation (SD). Prior to analysis, data were screened for outliers and evaluated for normality of distribution and homogeneity of variances to verify the assumptions required for parametric testing. All biological and chemical assays were performed in triplicate (*n* = 3), except the antifungal susceptibility assays (MIC and MFC), which were performed in duplicate (*n* = 2); the sample size (n) for each experiment is also indicated in the corresponding figure and table legends. Data were then subjected to one‐way analysis of variance (one‐way ANOVA), followed by Bonferroni's multiple‐comparison post hoc test (two‐sided), which includes the corresponding correction for multiple comparisons among the treatment groups. In some cases, the Student's t‐test was applied. A significance level of 5% (*α* = 0.05) was adopted, corresponding to a 95% confidence level, and results with *p* < 0.05 were considered statistically significant. Significance was denoted as follows: *p < 0.05, **p < 0.01, ***p < 0.001, and ****p < 0.0001. Statistical analyses were performed using GraphPad Prism software, version 5.0 (GraphPad Software Inc., San Diego, CA, USA).

## Author Contribution

**Tarsila da C. Silva**: investigation, formal analysis and writing, original draft. **Ari Sérgio de O. Lemos**: investigation (essential‐oil extraction and biological assays). **Caio Juan S. Fortes**: investigation (essential‐oil extraction). **Débora N. Eiriz** and **Lucas M. Duarte**: chromatographic analysis (GC‐MS). **Elaine S. Coimbra**: resources (cells and ethical approval for animal use, CEUA‐UFJF protocol 019/2026). **Lucas de A. Carvalho** and **Núbia B. Andrade**: investigation (antifungal assays). **Luciana M. Chedier** and **Rodrigo L. Fabri**: resources and supervision of the phytochemical and cell‐based/antifungal assays. **Selma R. de Paiva** and **Alessandra L. Valverde**: conceptualization, supervision and writing – review and editing. All authors read and approved the final version of the manuscript.

## Funding

The authors have nothing to report.

## Conflicts of Interest

The authors declare no conflicts of interest.

## Declaration of Generative AI and AI‐Assisted Technologies

During the preparation of this manuscript, the authors used Claude (model Opus 4.8; Anthropic) to assist with drafting, structuring and language editing of the text on the basis of data and content from the corresponding master's dissertation. No generative AI tool was used to create, alter or fabricate scientific data, figures or chemical structures. After using this tool, the authors reviewed and edited the content as needed and took full responsibility for the content of the publication.

## Data Availability

The data that support the findings of this study are available from the corresponding author upon reasonable request.
